# Inverse Association of Plasma Chromium Levels with Newly Diagnosed Type 2 Diabetes: A Case-Control Study

**DOI:** 10.3390/nu9030294

**Published:** 2017-03-17

**Authors:** Sijing Chen, Xiaoling Jin, Zhilei Shan, Shuzhen Li, Jiawei Yin, Taoping Sun, Cheng Luo, Wei Yang, Ping Yao, Kaifeng Yu, Yan Zhang, Qian Cheng, Jinquan Cheng, Wei Bao, Liegang Liu

**Affiliations:** 1 Department of Nutrition and Food Hygiene, Hubei Key Laboratory of Food Nutrition and Safety, School of Public Health, Tongji Medical College, Huazhong University of Science and Technology, Wuhan 430030, China; sijingchen19@sina.com (S.C.); xiaolingking@126.com (X.J.); shanzhilei3118@126.com (Z.S.); M201575207@hust.edu.cn (S.L.); M201575208@hust.edu.cn (J.Y.); suntaoping@163.com (T.S.); milkluobo@sina.com (C.L.); yw8278@hotmail.com (W.Y.); yaoping@mails.tjmu.edu.cn (P.Y.); bwinds@yeah.net (K.Y.); 2 Ministry of Education Key Lab of Environment and Health, School of Public Health, Tongji Medical College, Huazhong University of Science and Technology, Wuhan 430030, China; 3 Hubei Provincial Key Laboratory of Yeast Function, Yichang 443003, China; zhangyan@angelyeast.com (Y.Z.); chengqian@angelyeast.com (Q.C.); 4 Shenzhen Center for Disease Control and Prevention, Shenzhen 51805, China; cjinquan@szcdc.net; 5 Department of Epidemiology, College of Public Health, University of Iowa, Iowa City, IA 52242, USA; wei-bao@uiowa.edu

**Keywords:** chromium, type 2 diabetes, pre-diabetes

## Abstract

Chromium has long been known as an enhancer of insulin action. However, the role of chromium in the development of type 2 diabetes mellitus (T2DM) in humans remains controversial. The current study aimed to examine the associations of plasma chromium levels with T2DM and pre-diabetes mellitus (pre-DM). We conducted a case-control study involving 1471 patients with newly diagnosed T2DM, 682 individuals with newly diagnosed pre-DM, and 2290 individuals with normal glucose tolerance in a Chinese population from 2009 to 2014. Plasma chromium was measured by inductively coupled plasma mass spectrometry. Plasma chromium levels were lower in the T2DM and pre-DM groups than in the control group (median: 3.68 μg/L, 3.61 μg/L, 3.97 μg/L, respectively, *p* < 0.001). After adjustment for potential confounding factors, the odds ratios (95% confidence interval) for T2DM across increasing quartiles of plasma chromium levels were 1 (referent), 0.67 (0.55–0.83), 0.64 (0.51–0.79), and 0.58 (0.46–0.73), respectively (*p* for trend <0.001). The corresponding odds ratios (95% confidence interval) for pre-DM were 1 (referent), 0.70 (0.54–0.91), 0.67 (0.52–0.88), and 0.58 (0.43–0.78), respectively (*p* for trend < 0.001). Our results indicated that plasma chromium concentrations were inversely associated with T2DM and pre-DM in Chinese adults.

## 1. Introduction

Chromium, a putative essential micronutrient, has long been recognized as an enhancer of insulin action. Chromium has gained popularity as a nutritional supplement for diabetic and insulin-resistant subjects, but its role in the prevention and management of type 2 diabetes mellitus (T2DM) has not yet been established. Early clinical studies found that chromium deficiency might lead to glucose intolerance and insulin resistance [1,2]. Experimental studies in animal models have also demonstrated the potentially beneficial effects of chromium in alleviating diabetes, insulin resistance and lipid anomalies [3,4,5,6,7]. A recent study examining National Health and Nutrition Examination Survey (NHANES) data indicated that the risk of T2DM is lower in US adults taking chromium-containing supplements [8]. However, clinical trials evaluating chromium supplementation on glucose control have yielded conflicting results [9,10,11]. As a result, the routine use of chromium supplementation for glycemic control in patients with T2DM was not recommended due to insufficient evidence of effectiveness [12,13,14]. 

In addition, epidemiological data on chromium intake and the risk of T2DM are sparse, because of the difficulty in estimating dietary chromium due to its wide variability and low content in food sources. Hence, a sensitive and reliable biomarker for chromium intake is required in epidemiological studies. Several observational studies, with a small sample size, have reported lower chromium levels in patients with diabetes than in healthy controls [15,16]. To date, the dose-response relationship between chromium status and the risk of T2DM remains unknown. 

Pre-diabetes mellitus (pre-DM), characterized by impaired fasting glucose (IFG) and/or impaired glucose tolerance (IGT), is considered an important risk factor for the development of overt diabetes and cardiovascular disease [17,18,19,20]. Compared to individuals with normal glucose metabolism, patients with pre-DM have a five- to 15-fold higher risk of developing T2DM [21]. Likewise, few studies have focused on the relationship of chromium and pre-DM.

In this study with a large Chinese population, we aimed to examine the association of plasma chromium levels with T2DM and pre-DM. Furthermore, we assessed the dose-response relationship using a restricted cubic spline regression model.

## 2. Materials and Methods

### 2.1. Study Population

The study population consisted of 4443 patients: 1471 newly diagnosed T2DM patients, 682 newly diagnosed pre-DM patients, and 2290 individuals with normal glucose tolerance (NGT). Those with newly diagnosed pre-DM and T2DM were consecutively recruited from first-time patients attending the outpatient clinics of the Department of Endocrinology, Tongji Medical College Hospital, from January 2009 to December 2014. The healthy NGT individuals were recruited from an unselected population undergoing a routine health checkup in the same hospital, which were frequency-matched with T2DM by age (±5 years) and sex. The inclusion criteria of all participants were: 30 ≤ age < 70 years, body mass index (BMI) < 40 kg/m^2^, no history of diagnosed diabetes, not receiving pharmacological treatment for hyperlipidemia or hypertension, and not taking medication known to affect glucose tolerance or insulin secretion. Patients with clinically significant neurological, endocrinological or other systemic diseases, as well as acute illness and chronic inflammatory or infective diseases were excluded from the study. The definitions of T2DM, IFG, IGT, and NGT met the respective diagnostic criteria recommended by the World Health Organization in 1999 [22]. T2DM was diagnosed when fasting plasma glucose (FPG) ≥ 7.0 mmol/L and/or 2 h post-glucose load ≥11.1 mmol/L. Pre-DM was defined as IFG (FPG ≥ 6.1 and < 7.0 mmol/L and 2 h post-glucose load <7.8 mmol/L) and/or IGT (FPG < 7.0 mmol/L and 2 h post-glucose load ≥7.8 and <11.1 mmol/L). An FPG concentration <6.1 mmol/L and a plasma glucose concentration after 2-h oral glucose tolerance test <7.8 mmol/L was considered NGT. All the participants enrolled were of Chinese Han ethnicity. 

All subjects gave their informed consent for inclusion before they participated in the study. The study was conducted in accordance with the Declaration of Helsinki, and the protocol was approved by the Ethics Committee of Tongji Medical College.

### 2.2. Data Collection

Demographic, health status, and lifestyle data were obtained from the questionnaires, including sex, age, history of disease (hypertension and hyperlipemia), family history of diabetes, current smoking status, and alcohol drinking status. Current smoking and alcohol drinking were classified as yes or no. Anthropometric data including height (m), mass (kg) and blood pressure were measured with standardized techniques. BMI was calculated as mass divided by the square of height (kg/m^2^). 

### 2.3. Measurement of Glucose and Lipid Biomarkers

Blood samples were collected in all participants after an overnight fast of at least 10 h. All participants were given a standard 75 g glucose solution, and plasma glucose was measured at 0 and 2 h after administration during the oral glucose tolerance test. Fasting plasma insulin (FPI), total cholesterol, triglyceride, high-density lipoprotein cholesterol (HDLC), and low-density lipoprotein cholesterol (LDLC) was measured within 2 h, as described in our previous study [23]. Homoeostasis model assessment insulin resistance (HOMA-IR) score was computed using the following formula: FPI (μU/mL) × FPG (mmol/L)/22.5. The index of HOMA of β-cell function (HOMA-β) was calculated as [20 × FPI (μU/mL)]/[FPG (mmol/L) − 3.5].

### 2.4. Measurement of Plasma Chromium Concentrations

Plasma chromium concentrations were measured in the Ministry of Education Key Laboratory of Environment and Health and School of Public Health at Tongji Medical College of Huazhong University of Science & Technology, using inductively coupled plasma mass spectrometry (Agilent 7700 Series, Tokyo, Japan). Plasma samples were stored at −80 °C. For quality assurance, the certified reference material ClinChek no. 8883 and 8884 human plasma controls were used (certified concentration: 3.56 ± 0.89 μg/L, 11.1 ± 2.22 μg/L). The limit of detection (LOD) for chromium was 0.01 μg/L, and concentrations of plasma chromium levels below the LOD (0.7%) were imputed at LOD/$\sqrt{2}$. Quality control was performed (1 out of 20 samples), and the inter-assay and intra-assay coefficients of variation were <10% and <8%, respectively. 

### 2.5. Statistical Analysis

Descriptive statistics were calculated for all demographic and clinical characteristics of the study subjects, and summarized as mean with standard deviation (SD) for normally distributed data or median with interquartile range for non-normally distributed data. Comparisons between T2DM, pre-DM and controls were performed by χ^2^ test for categorical variables, or ANOVA for continuous variables, and then Dunnett post hoc tests were used to compare between groups.

For calculation of the odds ratio (OR) for T2DM and pre-DM, plasma chromium concentration was categorized in quartiles according to the NGT group: category 1, <3.04 μg/L; category 2, 3.04–3.96 μg/L; category 3, 3.96–5.22 μg/L, and category 4, >5.22 μg/L. Binary logistic regression (T2DM versus controls, or pre-DM versus controls) was used to assess the association of T2DM and pre-DM with plasma chromium concentrations. The ORs and 95% confidence intervals (CI) of T2DM and pre-DM were calculated between the quartiles of chromium using the lowest quartile as the reference category, and also by per 1-SD of log-transformed chromium as continuous variable. The ORs and 95% CIs were adjusted for known risk factors of T2DM including age, sex, BMI, family history of diabetes, hypertension, current smoking status (yes or no), and alcohol drinking (yes or no). Hosmer–Lemeshow tests were used to evaluate whether the model provided a good fit. Tests of linear trend across increasing chromium quartiles were conducted by assigning the median value to each quartile and treating it as a continuous variable. Additionally, Pearson correlation analysis was used to detect the correlation of log-transformed chromium concentrations with fasting glucose and insulin in the control group. A logarithmic transformation was used to improve the normality of plasma chromium distributions.

The dose-response relationship for T2DM was estimated by applying a restricted cubic spline regression model with 3 knots at the 5th (1.58 μg/L), 50th (3.82 μg/L) and 95th (8.43 μg/L) percentiles. To evaluate the consistency of the association between chromium and T2DM and pre-DM by participant characteristics, additional analyses were run, stratifying age (<45, ≥45), sex, BMI (<24, ≥24), current smoking status, current drinking alcohol status, and hypertension. Likelihood ratio tests were conducted to examine interactions. 

Statistical analyses were performed with SPSS for Windows, version 21.0 (SPSS Inc., Chicago, IL, USA). *P* values reported are two tailed, and values below 0.05 were considered statistically significant.

## 3. Results

### Patient Characteristics

Anthropometric and metabolic characteristics of the 4443 participants with NGT, pre-DM and T2DM are reported in Table 1. Plasma chromium concentrations were significantly decreased in the individuals with pre-DM and newly diagnosed T2DM compared with the controls (median: 3.97 μg/L in NGT, 3.61 μg/L in pre-DM, and 3.68 μg/L in T2DM, *p* < 0.001). Compared to control subjects, the individuals with pre-DM and newly diagnosed T2DM had a higher age, BMI and prevalence of family history of diabetes and hypertension. As expected, higher levels of FPG, post-glucose load after the 2 h oral glucose tolerance test (OGTT 2 h), FPI, HOMA-IR, and lower levels of HDLC were observed in newly diagnosed T2DM and pre-DM cases than in the controls.

**Figure 1:**
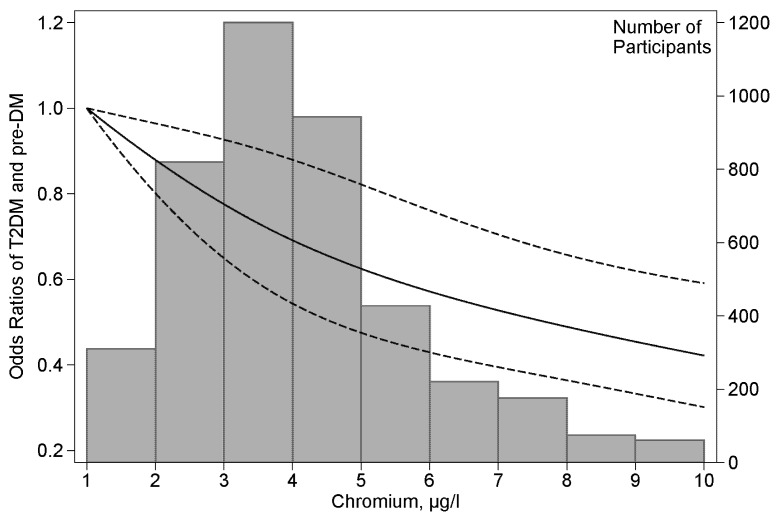
Odds ratios of T2DM and pre-DM by plasma chromium concentrations. Lines represent odds ratios (95% CIs) based on restricted cubic splines for plasma chromium concentrations with knots at the fifth, 50th, and 95th percentiles. Odds ratios were estimated using a logistic regression model after adjustments for age, sex, BMI, current smoking status, current alcohol drinking status, family history of diabetes and hypertension. Bars represent the numbers of participants; nine equally sized bins were selected from the second to the 98th percentiles of chromium distribution.

Table 2 presents ORs for T2DM and pre-DM associated with the levels of plasma chromium concentration which are categorized into quartiles according to their distribution in the control subjects. After multivariable adjustment for age, sex, BMI, lifestyle covariates, family history of diabetes, and hypertension, the ORs (95% CIs) for T2DM from the lowest to the highest quartiles were 1 (reference), 0.67 (0.55–0.83), 0.64 (0.51–0.79), and 0.58 (0.46–0.73), respectively (*p* for trend <0.001). The adjusted ORs of pre-DM were similar to those of T2DM. Moreover, similar results were obtained when combining the T2DM group and the pre-DM group. When the plasma chromium concentration was considered as a continuous variable, the overall OR (95% CI) of having T2DM or pre-DM was 0.88 (0.82–0.94) per 1 SD increment of the log-transformed chromium concentration. A positive linear dose-response relationship for T2DM was evident in the cubic spline regression model (Figure 1). Furthermore, Pearson correlation analysis showed a significant and inverse correlation of log-transformed chromium concentrations with fasting glucose (coefficient = −0.166, *p* < 0.001) in the control group, but no significant correlation with fasting insulin.

The chromium-diabetes association was not significantly different by sex, BMI, smoking, or history of hypertension (Table 3). Significant interactions were observed between chromium and age, and chromium and alcohol drinking.

## 4. Discussion

In this case-control study, inverse associations were found between plasma chromium concentrations and the prevalence of newly diagnosed type 2 diabetes and pre-diabetes among Chinese adults. The chromium-diabetes association was not appreciably changed by adjusting for age, sex and BMI, current smoking status, current alcohol drinking status, family history of diabetes and hypertension. These results were consistent in the stratified analyses.

Previous studies reporting plasma chromium concentrations in large populations were sparse. The median concentration of plasma chromium in our population was 3.96 μg/L (interquartile range: 3.04–5.22 μg/L), higher than the previously published studies, which varied from 0.2 to 0.86 μg/L [16,24,25]. Currently, there is no international acceptable value or range for the plasma chromium concentration in the general population. The main sources of chromium exposure among the general population are foods and contamination. Chromium coming from foods is very low in its trivalent form [26]. Dietary intake of chromium from Asian diets ranged from 59.9 to 224 μg per day [27]. Nevertheless, large amounts of chromium leach into food cooked in stainless steel [28]. Chromium exposure from industrial pollution is mainly in hexavalent form, via air and water. China’s anthropogenic chromium emissions showed a dramatic increase from 1990 to 2009. Coal and oil combustion, the metal fabrication industry and the leather tanning sector were the dominant sources of chromium emissions [29]. In addition, tobacco smoking and drinking beer are additional small but important sources of non-occupational chromium exposure [30,31]. In the present study, it was shown that the median concentration of plasma chromium was higher in smokers and alcohol drinkers than in their non-smoking and non-alcohol-drinking counterparts as well.

The results on the association between plasma chromium and T2DM are in accordance with the previous epidemiological studies, although such studies are very limited. Two studies indicated that chromium levels were significantly lower in patients with diabetes compared to the control [15,16]. However, these two studies involved a very small number of cases (*n* = 20 and *n* = 53, respectively). The Coronary Artery Risk Development in Young Adults (CARDIA) trace element study found that chromium levels were inversely correlated with HOMA-IR among young US adults [32]. Numerous studies have evaluated the short-term effects of chromium supplementation on carbohydrate and lipid metabolism parameters in humans, but yielded mixed and controversial results. Previous meta-analyses found no significant effect of chromium supplementation on glucose concentrations in non-diabetic individuals, and were inconclusive with regard to glycemia control among diabetic patients [9,10,11]. The poor study quality, high level of heterogeneity, lack of consensus on the assessment of chromium status, and no information on the bioavailability of the different forms of chromium were limiting factors of these studies. In this study, significant interactions were found between the plasma chromium concentration and age, as well as alcohol intake. The interaction has not been assessed in previous studies and may warrant confirmation in further studies.

In contrast to conflicting results in human studies, animal and in vitro studies have consistently demonstrated a beneficial effect of chromium in alleviating diabetes and insulin resistance. Two molecules, low-molecular-weight chromium-binding substance (LMWCr, also called chromodulin) and chromate, have been suggested as the biologically active form of chromium. The underlying molecular mechanisms are that chromium may up-regulate insulin-stimulated insulin signal transduction. Chromium may enhance the kinase activity of IR-β [33,34], increase the activity of downstream effectors of insulin signaling PI3K and Akt [3], and enhance Glut4 translocation to the cell surface [5,35]. In addition, chromium blunts the negative regulators of insulin signaling, such as PTP-1B [3], c-Jun N-terminal kinase (JNK) and IRS-1 serine phosphorylation [4]. Chromium may also enhance AMPK activity transiently to increase glucose uptake [36]. Furthermore, chromium alleviates endoplasmic reticulum (ER) stress within the cells [4], although the exact mechanism is unclear. The potential effect of chromium in attenuating oxidative stress has also been addressed [37].

The strengths of our study included the large number of participants and objectively measured plasma chromium levels. The subjects with T2DM were confined to the newly diagnosed and no medication because anti-diabetic drugs may alter the status of chromium metabolism, and large losses of chromium over more than two years’ diabetes duration may change the chromium homeostasis [38]. Moreover, the association between plasma chromium and pre-DM was assessed, which was consistent with the results of T2DM. In addition, chromium levels in plasma were measured using the state-of-the-art ICP-MS method and plasma chromium is considered a reliable objective biomarker for chromium exposure [39].

A few limitations need to be considered. First, the case-control nature of this study does not allow us to infer any causality and address the temporal relationship between plasma chromium and the development of T2DM. Second, trivalent chromium was not differentiated from hexavalent chromium in the plasma measurement. Trivalent chromium is suggested to be beneficial and hexavalent chromium is toxic to human health [40]. Thus, the combination of these two forms may attenuate any association that may exist between trivalent chromium and T2DM and its components. Nevertheless, no objective biomarkers of trivalent chromium are available for large-scale epidemiological study. Third, the lack of information on physical activity, education level, and the other unknown or unmeasured factors might also confound the results. Additionally, subjects with proteinuria were not excluded, which may have an effect on chromium loss. Moreover, regarding the lack of information on the background diet and the levels of other minerals purported to affect T2DM, notably magnesium but also zinc. Further investigation on their relationship and/or interaction with chromium and glucose homeostasis is needed. Finally, the generalizability of the findings may be limited since all participants were of Chinese Han ethnicity. However, a homogenous ethnic background may reduce residual confounding factors from unmeasured genetic and cultural variability.

## 5. Conclusions

Our study demonstrated an inverse association between plasma chromium concentrations and T2DM and pre-diabetes in a Chinese population. Further studies are warranted to confirm our findings in prospective cohorts and to elucidate the potential mechanisms underlying the relationship between chromium and T2DM. 

## Figures and Tables

**Table 1:** Anthropometric and metabolic characteristics of NGT, pre-DM and T2DM groups.

Parameters	NGT (*n* = 2290)	Pre-DM (*n* = 682)	T2DM (*n* = 1471)	*p* Value	
				Pre-DM vs. NGT	T2DM vs. NGT
Male sex, *n* (%)	1481 (64.67%)	409 (59.97%)	936 (63.63%)	0.022	0.514
Age (year)	46.93 (10.28)	49.47 (9.93)	48.39 (9.60)	<0.001	<0.001
BMI (kg/m^2^)	23.24 (3.06)	24.80 (3.28)	25.20 (3.36)	<0.001	<0.001
Current smoker, *n* (%)	924 (40.34%)	193 (28.30%)	562 (38.21%)	<0.001	0.322
Current drinker, *n* (%)	828 (36.16%)	210 (30.79%)	537 (36.51%)	0.028	0.493
Family history of diabetes, *n* (%)	239 (10.44%)	138 (20.23%)	335 (22.77%)	<0.001	<0.001
Hypertension, *n* (%)	542 (23.67%)	242 (35.48%)	524 (35.62%)	<0.001	<0.001
FPG (mmol/L)	5.19 (0.75)	6.11 (0.54)	9.04 (3.21)	<0.001	<0.001
OGTT 2 h (mmol/L)	6.41 (0.95)	8.81 (1.36)	16.79 (4.93)	<0.001	<0.001
FPI (mmol/L)	6.85 (4.66)	9.23 (5.86)	10.65 (8.34)	<0.001	<0.001
HOMA-IR	1.67 (1.18)	2.50 (1.72)	3.85 (3.01)	<0.001	<0.001
HOMA-β	71.34 (57.45)	73.87 (51.78)	46.26 (61.67)	0.612	0.879
TC (mmol/L)	4.40 (1.23)	4.41 (1.53)	4.42 (1.58)	0.069	0.909
TG (mmol/L)	1.30 (0.78)	1.26 (0.88)	1.40 (1.02)	0.831	<0.001
HDLC (mmol/L)	1.35 (0.34)	1.18 (0.53)	1.12 (0.55)	<0.001	<0.001
LDLC (mmol/L)	2.37 (0.97)	2.43 (1.14)	2.44 (1.32)	0.344	0.090
Chromium (μg/L)	3.96 (2.19)	3.61 (1.77)	3.68 (1.97)	<0.001	<0.001

Data were presented as number (percentage) for categorical data, mean (SD) for normally distributed data, or median (interquartile range) for non-normally distributed data. Abbreviations: FPG: fasting plasma glucose; FPI: fasting plasma insulin; HDLC: high-density lipoprotein cholesterol; HOMA-IR: homeostasis model assessment of insulin resistance; HOMA-β: homeostasis model assessment of beta cell function; LDLC: low-density lipoprotein cholesterol; NGT: normal glucose tolerance; OGTT 2 h: post-glucose load after 2 h oral glucose tolerance test; Pre-DM: pre-diabetes mellitus; T2DM: type 2 diabetes mellitus; TC: total cholesterol; TG: triglyceride.

**Table 2:** Association of plasma chromium concentrations with T2DM and pre-DM.

Variables	Quartiles of Plasma Chromium Concentrations				Per 1 SD of Log-Plasma Chromium	*p* Value for Trend
	1 (Lowest)	2	3	4 (Highest)		
	<3.04 μg/L	3.04–3.96 μg/L	3.96–5.22 μg/L	>5.22 μg/L		
T2DM/NGT						
Crude	1	0.74 (0.62–0.89)	0.74 (0.62–0.88)	0.56 (0.46–0.67)	0.86 (0.81–0.92)	<0.001
Model 1	1	0.70 (0.58–0.85)	0.70 (0.58–0.85)	0.53 (0.43–0.66)	0.86 (0.80–0.92)	<0.001
Model 2	1	0.67 (0.55–0.83)	0.64 (0.51–0.79)	0.58 (0.46–0.73)	0.88 (0.82–0.95)	<0.001
Pre-DM/NGT						
Crude	1	0.83 (0.66–1.05)	0.78 (0.62–0.98)	0.50 (0.38–0.64)	0.88 (0.82–0.95)	<0.001
Model 1	1	0.73 (0.57–0.93)	0.69 (0.54–0.89)	0.52 (0.39–0.69)	0.88 (0.81–0.96)	<0.001
Model 2	1	0.70 (0.54–0.91)	0.67 (0.52–0.88)	0.58 (0.43–0.78)	0.91 (0.83–0.99)	<0.001
T2DM&Pre-DM/NGT						
Crude	1	0.77 (0.65–0.90)	0.75 (0.64–0.88)	0.54 (0.45–0.64)	0.86 (0.81–0.91)	<0.001
Model 1	1	0.71 (0.60–0.85)	0.71 (0.59–0.84)	0.53 (0.44–0.64)	0.86 (0.80–0.91)	<0.001
Model 2	1	0.68 (0.57–0.82)	0.66 (0.54–0.79)	0.58 (0.47–0.71)	0.88 (0.82–0.94)	<0.001

Model 1: adjusted for age, sex and BMI; Model 2: additionally adjusted for current smoking status, current alcohol drinking status, family history of diabetes and hypertension. Abbreviations: NGT: normal glucose tolerance; Pre-DM: pre-diabetes mellitus; T2DM: type 2 diabetes mellitus.

**Table 3:** Adjusted ORs for T2DM and pre-DM of plasma chromium in subgroups.

Participant	Quartiles of Plasma Chromium Concentrations				Per 1 SD Log-Plasma Cr	*p* for Interaction
	1 (Lowest)	2	3	4 (Highest)		
	<3.04 μg/L	3.04–3.96 μg/L	3.96–5.22 μg/L	>5.22 μg/L		
Subgroup						
Age						<0.001
<45	1	1.00 (0.74–1.36)	1.08 (0.75–1.23)	0.45 (0.33–0.62)	0.88 (0.80–0.97)	
≥45	1	0.56 (0.44–0.71)	0.47 (0.37–0.61)	0.85 (0.63–1.13)	0.90 (0.80–1.00)	
Sex						0.706
Women	1	0.57 (0.42–0.81)	0.59 (0.42–0.81)	0.55 (0.39–0.79)	0.90 (0.80–1.01)	
Men	1	0.76 (0.60–0.95)	0.69 (0.55–0.88)	0.59 (0.45–0.76)	0.87 (0.80–0.95)	
BMI						0.424
<24	1	0.56 (0.43–0.74)	0.59 (0.45–0.77)	0.56 (0.41–0.74)	0.86 (0.79–0.95)	
≥24	1	0.82 (0.63–1.06)	0.75 (0.57–0.97)	0.62 (0.47–0.83)	0.91 (0.82–1.00)	
Smoking						0.195
No	1	0.69 (0.55–0.86)	0.68 (0.54–0.86)	0.69 (0.53–0.90)	0.93 (0.85–1.01)	
Yes	1	0.68 (0.49–0.95)	0.61 (0.45–0.83)	0.44 (0.31–0.61)	0.81 (0.72–0.71)	
Drinking alcohol						0.026
No	1	0.69 (0.55–0.87)	0.72 (0.57–0.91)	0.72 (0.56–0.93)	0.96 (0.88–1.05)	
Yes	1	0.71 (0.51–0.99)	0.55 (0.40–0.76)	0.38 (0.26–0.54)	0.74 (0.65–0.84)	
Hypertension						0.125
No	1	0.66 (0.53–0.82)	0.71 (0.57–0.89)	0.53 (0.41–0.67)	0.87 (0.80–0.94)	
Yes	1	0.75 (0.53–1.07)	0.58 (0.41–0.82)	0.70 (0.48–1.02)	0.91 (0.79–1.04)	
Overall	1	0.68 (0.57–0.82)	0.66 (0.54–0.79)	0.58 (0.47–0.71)	0.88 (0.82–0.94)	

Adjusted for age, sex, BMI, current smoking status, current alcohol drinking status, family history of diabetes and hypertension.
